# Deep First Formal Concept Search

**DOI:** 10.1155/2014/275679

**Published:** 2014-08-31

**Authors:** Tao Zhang, Hui Li, Wenxue Hong, Xiamei Yuan, Xinyu Wei

**Affiliations:** ^1^School of Information Science and Engineering, Yanshan University, Qinhuangdao 066004, China; ^2^School of Electrical Engineering, Yanshan University, Qinhuangdao 066004, China; ^3^College of Foreign Languages, Yanshan University, Qinhuangdao 066004, China

## Abstract

The calculation of formal concepts is a very important part in the theory of formal concept analysis (FCA); however, within the framework of FCA, computing all formal concepts is the main challenge because of its exponential complexity and difficulty in visualizing the calculating process. With the basic idea of Depth First Search, this paper presents a visualization algorithm by the attribute topology of formal context. Limited by the constraints and calculation rules, all concepts are achieved by the visualization global formal concepts searching, based on the topology degenerated with the fixed start and end points, without repetition and omission. This method makes the calculation of formal concepts precise and easy to operate and reflects the integrity of the algorithm, which enables it to be suitable for visualization analysis.

## 1. Introduction 

FCA, a branch of lattice theory, presented by Ganter and Wille [1] in 1982, is a discipline that concerns the mathematical concepts and conceptual hierarchies. As a powerful tool for data analysis and knowledge processing, FCA has been widely applied in date mining [2, 3], web search [4, 5], software engineering [6, 7], ontology analysis [8, 9], and so forth and still has a great potential value in applications.

Computing all formal concepts and the concept lattice is the most basic issue that has been investigated by many domestic and foreign researchers from different angles. Various forms of algorithms in concepts computing and concept lattice generation can be generally divided into three categories: batch processing algorithm [10–14], progressive algorithm [15, 16], and parallel algorithms [17, 18]. The basic idea of batch processing algorithm is to form the edges according to the presubsequent relationship among all concepts generated and then constructs the concept lattice. Progressive algorithm intends to initialize the concept lattice to empty firstly and then updates the concepts according to the different results of the intersection operation between the new concept which is to join to the lattice and all the concepts of the current concept lattice. Parallel algorithm intends to split the formal context into some subcontexts, based on which to construct sublattices, and then makes the appropriate merge operations. Besides, progressive algorithm [19, 20] and parallel algorithms [21, 22] work very effectively in the calculation of concepts.

However, the structure of concept lattice is relatively complex and the relationship among attributes of formal context cannot be visually represented. Attribute topology [23–25] presented by Zhang et al. is a new method to represent the formal context. Different with the traditional representation of formal context, attribute topology is represented by the weighted graph, with the attributes as the vertices and inclusion relation between attributes as the weights, which performs the coupling relationship and the coupling strength between attributes intuitively. Attribute topology, providing a new method to represent formal context, not only performs the association and the strength of association simply and intuitively but also has a one-to-one relationship with formal context [23].

Based on the new representation, Zhang et al. present a calculation algorithm of formal concepts by attribute topology [24]. The method, firstly, intends to construct the subtopology successively according to the number of connections with the top-attributes, as the core of the subtopology, respectively, with the order from more to less. Then, all extensions in every subtopology are achieved after the arrangement and operation of all the available object sets according to the association and correlation intensity, and all concepts in every subtopology are generated with the corresponding intensions, that is, all concepts in the formal context. This method calculating concept by the attribute topology not only provides a new idea for the calculation of formal concepts but also enables the calculation simple and easy to operate. However, the method is not suitable for visualization analysis due to the subtopology that is achieved by splitting the whole topology. Secondly, it is not available for the concepts' calculation in the formal context of large-scale date due to the poor visibility.

Global formal concepts searching of attribute topology is proposed in this paper with the basic idea of Depth First Search. This method, firstly, adds two vertices, global start and end points, and edges to the original attribute topology, degenerating it into the topology with the fixed start and end points and then, limited by the constraints and calculation rules, explores and backtracks the vertices, sorted formerly, repetitively, completing the traversal of paths. All formal concepts are achieved by the path traversing between the global start and end points. This method constructs the topology into a complete whole, avoiding the decomposition process of the whole topology, and reflects the integrity of the algorithm. Calculation process is demonstrated intuitively in the process of traversing paths, reflecting the good visibility.

## 2. Basic Notions

### 2.1. The Basis of Formal Context

Formal context, which acts as the research object and date representation, is an important basic aspect in FCA. Here are a few notions about formal context [1].

**Definition 1 .**

A formal context can be expressed as *K*≔(*G*, *M*, *I*) which is composed of two sets *G* and *M* and the relation *I* between them. Where *G* is a set of objects, *M* is a set of attributes, and *I* is an object-attribute relation.

**Definition 2 .**

Let *K*≔(*G*, *M*, *I*) be a formal context, *A*⊆*G*, *B*⊆*G*; let

(1) $$\begin{array}{c} f\left(A\right)=\left\{m\in M\;|\;\forall g\in A,\left(g,m\right)\in I\right\},\\ g\left(B\right)=\left\{g_{0}\in G\;|\;\forall m\in B,\left(g_{0},m\right)\in I\right\}. \end{array}$$

If *f*(*A*) = *B* and *g*(*B*) = *A*, then the pair (*A*, *B*) is a formal concept. The sets *A* and *B* are the extension and intension of (*A*, *B*), respectively. The set of all concepts in *K*≔(*G*, *M*, *I*) is denoted by *β*(*G*, *M*, *I*) or *β*(*K*).

**Definition 3 .**

The concept, with the whole attributes as the intension and the corresponding object set as the extension (called global concept of whole attribute) or the whole objects as the extension and the corresponding attribute set as the intension (called global concept of whole object), is called global concept. There exist only two global concepts in *K*≔(*G*, *M*, *I*), that is, (*G*, *f*(*G*)) and (*g*(*M*), *M*).

Concept lattice, the core date structure of FCA, is a complete lattice denoted by all concepts and the generalization-specialization relationships between them. Hasse diagram, equipped with the partial order of concept lattice simply and effectively, is the best way and common method to represent the concept lattice, which can express the relationships between all concepts intuitively and integrally.

Every vertex in Hasse diagram is a concept and all vertices respect the whole concepts in formal context. The entire Hasse diagram is constructed by all the concepts together with the order inclusion relation between two formal concepts, and each layer is arranged in descending order of the extension (ascending order of the intension).

Formal contexts mentioned in this paper are all simplified contexts [23–25].

### 2.2. The Basis of Attribute Topology

From the perspective of graph theory, the attribute topological representation is the weighted graph which concerns the relationship among attributes. So it can use the storage method of graph, that is, adjacency matrix sequence. In [23, 24], attribute topology is represented by adjacency matrix from the perspective of inclusion relation of attributes.

Suppose *T* = (*V*, Edge) is the attribute topological representation for formal context *K*≔(*G*, *M*, *I*). *V* = *M* is the set of vertices in attribute topology. Edge is the set of weights on edges in attribute topology.

In this paper, adjacency matrix, induced in [23, 24], is streamlined, and the adjacency matrix modified is

(2) Edge(mi,mj) ={Φ,g(mi)∩g(mj) =Φorg(mi)⊂g(mj)g(mi)∩g(mj),others.

Here are a few notions about attribute topology [22–25].

**Definition 4 .**

Global attribute is the attribute that possess all objects. In the formal context *K*∶ = (*G*, *M*, *I*), if *m* ∈ *M* and *g*(*m*) = *G*, then attribute *m* is a global attribute. Correspondingly, global object is the object that possesses all attributes.

**Definition 5 .**

Empty attribute is the attribute that does not possess any object in formal context. In *K*≔(*G*, *M*, *I*), if *m* ∈ *M* and *g*(*m*) = Φ, then attribute *m* is an empty attribute. Correspondingly, empty object is the object that does not possess any attribute.

According to the lattice theory, the global objects and global attributes only emerge on the top and at the bottom of the concept lattice, and they will not influence the structure of the concept lattice, so the global objects and global attributes can be reduced in concept lattice [24]. Empty objects and empty attributes which have no association with any other attributes and objects are independent existence that have no influence on the process of calculating concepts.

**Definition 6 .**

In attribute topology, if any Edge(*m*_*i*_, *m*_*j*_) ≠ Φ satisfying Edge(*m*_*j*_, *m*_*i*_) ≠ Φ, then *m*_*j*_ attribute is called top-attribute [21].

Edges connected to the top-attributes in attribute topology are bidirectional or unidirectional pointing to the outside.

**Definition 7 .**

In *K*≔(*G*, *M*, *I*), *m*_*i*_ ∈ *M*, let *N* be a nonempty set, for ∀*m*_*i*_ ∈ *N* ⊂ *M* − {*m*_*i*_} satisfying *g*(*m*_*i*_) ⊂ *g*(*m*_*j*_); then *m*_*i*_ attribute is the child-attribute of *m*_*j*_ attribute and *m*_*j*_ attribute is the father-attribute of *m*_*i*_ attribute [25].

**Theorem 8 .**

In *K*≔(*G*, *M*, *I*), if *A* ⊂ *M* and for ∀*a*_*i*_ ∈ *A*, satisfying *g*(*m*) ⊂ *g*(*a*_*i*_), that is, *m* attribute is the child-attribute of some attributes; *m* attribute may possess several father-attributes.

*F*(*m*) is induced by all father-attributes of *m* child-attribute.

**Theorem 9 .**

A top-attribute is definitely not the child-attribute.

## 3. Global Formal Concepts Searching of Attribute Topology

Global formal concepts searching of attribute topology are induced in the paper in order to construct the relationship between concepts while calculating all the concepts. Limited by the constraints and calculation rules, a series of paths are formed through exploring and backtracking the vertices of attributes sorted formerly while computing all formal concepts, in which removes the relevant edges of topology simultaneously, based on the topology degenerated with the fixed start and end points.

### 3.1. Degeneration of Attribute Topology

According to [25], all attributes in formal context are divided into the top-attributes and child-attributes. Meanwhile, [24] puts the top-attributes as the core to decompose the topology into some subtopologies.

This paper, firstly, establishes the global attribute Ψ and the empty attribute *E* from the global point of view. Then, without changing the basic structure of the topology, the topology model, possessing the global start and end points, is constructed. This model, denoted by topological degeneration model, represents the degeneration of topology.

Topological degeneration model mentioned above is described as follows: vertices Ψ and *E* (*g*(Ψ) = *G*, *g*(*E*) = Φ) are added to the original topology, with Ψ as the global start point and *E* as the global end point. Suppose that sets *A*, *B*⊆*M*, for ∀*m*_*i*_ ∈ *A*, generate the unidirectional edges 〈Ψ, *m*_*i*_〉 and let Edge(Ψ, *m*_*i*_) = *g*(*m*_*i*_); for ∀*m*_*j*_ ∈ *B*, let 〈*m*_*j*_, *E*〉 = End, where End is the terminal symbol and is represented by unidirectional edge when drawing in order to describe the model uniformly.

The establishment of the topological degeneration model can be divided into the following two cases.

 (*1) No Existence of Child-Attributes*. Then let *A* = *M* and *B* = *M*; that is, for ∀*m* ∈ *M*, let Edge(Ψ, *m*) = *g*(*m*), 〈*m*, *E*〉 = End.

(*2) Existence of Child-Attributes.* Let *A* be the set of top-attributes and *B* the set of child-attributes; that is, *A* ∪ *B* = *M* and *A*∩*B* = Φ.

**Figure 1:**
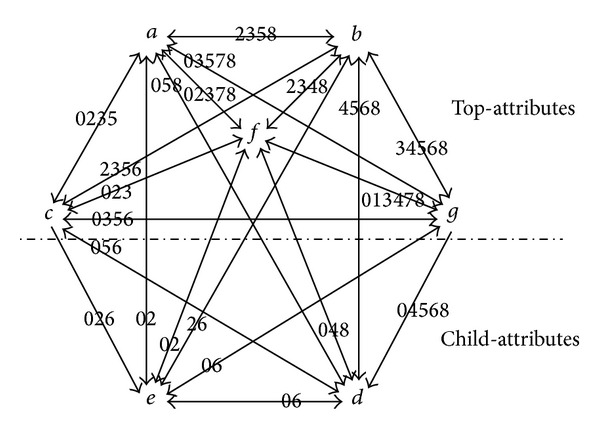
The attribute topology of the formal context from Table 1.

The topological degeneration model is established by the following example: Table 1 shows a formal context that contains child-attributes. The attribute topology of the formal context listed in Table 1 is presented in Figure 1.

**Figure 2:**
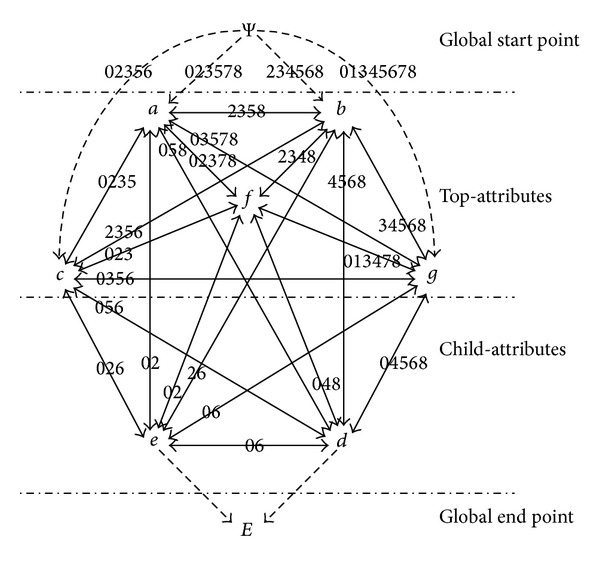
The topological degeneration model from the topology shown in Figure 1.

In the formal context (see Table 1), {*a*, *b*, *c*, *g*, *f*} is the set of top-attributes and {*d*, *e*} is the set of child-attributes. The degeneration of the topology (see Figure 1) is shown as follows: vertices Ψ and *E* are added to the original topology and let *A* = {*a*, *b*, *c*, *g*, *f*}, *B* = {*d*, *e*}, then construct the unidirectional edges from Ψ to every element in set *A* and unidirectional edges from every element in set *B* to *E*. The topology degenerated is presented in Figure 2.

The degeneration of the topology (see Figure 2) is presented by the newly added vertices Ψ, *E*, and the dashed lines with an arrow, according to the comparison of Figures 1 and 2. A complete whole of topology is achieved after the degeneration, by constructing the unidirectional edges from global start point to related vertices and unidirectional edges from related vertices to global end point.

The degeneration of the topology (see Figure 2) that contains child-attributes is actually achieved by constructing the unidirectional edges from global start point to all top-attributes and unidirectional edges from all child-attributes to the global end point, while, in terms of the topology without child-attributes, its degeneration is realized by constructing the unidirectional edges from global start point to all attributes and unidirectional edges from all attributes to the global end point.

Above analysis shows that the added two vertices and edges have no influence on the original association and correlation intensity between attributes. That is, the structure of the original topology has no changes, still containing the association and correlation intensity between all attributes and objects. The association and correlation intensity required in calculating the concepts are not damaged, and subsequent calculation of concepts based on the degenerated topology has not been affected.

From the perspective of graph theory, a complete diagram with fixed start and end points, suitable for the following path searching, is achieved by the degeneration.

### 3.2. Algorithm Description

The traversal on path, the case of exploring and backtracking vertices, is fundamentally carried from the global start point Ψ and ends with traversing all paths between Ψ and *E*. The certificates for exploring and backtracking vertices are provided by the constraints and calculation rules.

#### 3.2.1. Preliminaries

(*1) Sorted Vertices*. Suppose that *M* is the set of all attributes in attribute topology, the set of all top-attributes *A*⊆*M*, and the set of all child-attributes *B*⊆*M*.

For ∀*m*_*i*_ ∈ *C*⊆*M*, let num(*m*_*i*_) = #{*n*∣Edge(*n*, *m*_*i*_) ≠ Φ or Edge(*m*_*i*_, *n*) ≠ Φ, *n* ∈ *M* − *m*_*i*_}. #{·} represents the number of elements in the collection.

**Definition 10 .**

Let *C* be a nonempty set and *C*⊆*M*. A mapping *T* : *C* → *C* satisfies the following:

1. *C* ↦ *T*(*C*)≜*C* ^*T*^ = {*c*_1_, *c*_2_,…, *c*_*i*_∣*c*_*k*_ ∈ *C*, *k* ∈ [1, *i*]};
2. num(*c*_1_) ≤ num(*c*_2_) ≤  ⋯≤  num(*c*_*i*_).

According to Definition 10, *A* ^*T*^, an ordered set, is the result of reordering all the elements in set *A*. *B* ^*T*^ is the result of reordering elements in *B* by the same ruler.

Let *M*_(*A*,*B*)_^*T*^ = {Ψ, *A* ^*T*^, *B* ^*T*^, *E*}; that is, *M* ^*T*^_(*A*,*B*)_ = {Ψ, *m*_1_, *m*_2_,…, *m*_*i*_, *n*_1_, *n*_2_,…, *n*_*j*_, *E*}.

According to the above analysis, *M*_(*A*,*B*)_^*T*^ is an ordered set after reordering the set of all attributes added to the global start and end points. The subsequent exploring and backtracking of vertices are based on *M* ^*T*^_(*A*,*B*)_, in which the global start point is followed by a series of ordered top-attributes followed by ordered child-attributes and the global end point in the last.

In the formal context shown in Table 1, *M* = {*a*, *b*, *c*, *d*, *e*, *f*, *g*}, the set of top-attributes *A* = {*a*, *b*, *c*, *g*, *f*}, and set of child-attributes *B* = {*d*, *e*}. According to the mapping *T*, *A* ^*T*^ = {*a*, *b*, *c*, *g*, *f*}, *B* ^*T*^ = {*d*, *e*}, and *M* ^*T*^_(*A*,*B*)_ = {Ψ, *a*, *b*, *c*, *g*, *f*, *d*, *e*, *E*}.

(*2) Improved Description of Child-Attribute*. In order to calculate concepts, some related properties of child-attribute are introduced in this section.

**Definition 11 .**

*P* ^*a*^ (empty or nonempty) is a set that follows the following:

1. *a* is a child-attribute;
2. #*P* ^*a*^ ≥ 1 or #*P* ^*a*^ = 0;
3. *P* ^*a*^ = ⋃_*i*∈*T*_ *P*_*i*_^*a*^,

where ∀*P*_*i*_^*a*^ ∈ *P* ^*a*^ holds the following conditions:

1. *P*_*i*_^*a*^ is a nonset of attributes;
2. #*P*_*i*_^*a*^ ≥ 2;
3. for ∀*m* ∈ *P*_*i*_^*a*^, *g*(*am*) = *O*_*i*_^*a*^ ⊂ *g*(*a*) where *O*_*i*_^*a*^ is a nonempty set of objects corresponding to *P*_*i*_^*a*^;
4. for ∀*n* ∈ *M* − *P*_*i*_^*a*^, then *g*(*an*) ≠ *O*_*i*_^*a*^;
5. if #*P* ^*a*^ > 1, for ∀*P*_*j*_^*a*^ ∈ *P* ^*a*^(*j* ≠ *i*), satisfying *P*_*j*_^*a*^ ≠ *P*_*i*_^*a*^ and *O*_*j*_^*a*^ ≠ *O*_*i*_^*a*^.

**Theorem 12 .**

If #*P* ^*a*^ ≠ 0, for ∀*P*_*i*_^*a*^ ∈ *P* ^*a*^, *M*_*T*_ = {*m*_*i*_∣*m*_*i*_ ∈ *a* ∪ {*P*_*i*_^*a*^}} must be a complete polygon.

**Proof**

For ∀*P*_*i*_^*a*^ ∈ *P* ^*a*^, ∀*m* ∈ *P*_*i*_^*a*^, then

(3) $$\begin{array}{c} g\left(a\right)\cap g\left(m\right)=O_{i}^{a}\neq \Phi . \end{array}$$

For ∀*m*_*i*_, *m*_*j*_ ∈ *P*_*i*_^*a*^, then

(4) $$\begin{array}{c} g\left(a\right)\cap g\left(m_{i}\right)=O_{i}^{a}g\left(a\right)\cap g\left(m_{j}\right)=O_{i}^{a}. \end{array}$$

Also

(5) g(a)∩g(mi)∩g(mj) =(g(a)∩g(mi))∩(g(a)∩g(mj))

combined with Formulae (4) and (5):

(6) $$\begin{array}{c} g\left(a\right)\cap g\left(m_{i}\right)\cap g\left(m_{j}\right)=O_{i}^{a}\neq \Phi . \end{array}$$

Also

(7) $$\begin{array}{c} g\left(a\right)\cap g\left(m_{i}\right)\cap g\left(m_{j}\right)\subseteq g\left(m_{i}\right)\cap g\left(m_{j}\right). \end{array}$$

Simultaneous Formulae (6) and (7) will get

(8) $$\begin{array}{c} g\left(m_{i}\right)\cap g\left(m_{j}\right)\neq \Phi \end{array}$$

combined with Formulae (4) and (8):

(9) $$\begin{array}{c} g\left(m_{i}\right)\cap g\left(m_{j}\right)\neq \Phi \;\left(i\in T,\;\;j\in T\right). \end{array}$$

So *M*_*T*_ = {*m*_*i*_∣*m*_*i*_ ∈ *a* ∪ {*P*_*i*_^*a*^}} is a complete polygon.

**Theorem 13 .**

If  #*P* ^*a*^ ≠ 0, for ∀*P*_*i*_^*a*^, *P*_*j*_^*a*^ ∈ *P* ^*a*^, satisfying *P*_*i*_^*a*^∩*P*_*j*_^*a*^ = Φ.

**Proof**

According to Definition 11, for ∀*P*_1_^*a*^, *P*_2_^*a*^ ∈ *P* ^*a*^, there exists *O*_1_^*a*^, *O*_2_^*a*^, respectively, and *O*_1_^*a*^ ≠ *O*_2_^*a*^:

 for ∀*m*_*i*_ ∈ *P*_1_^*a*^, *g*(*m*_*i*_ *a*) = *O*_1_^*a*^;
 similarly, for ∀*m*_*j*_ ∈ *P*_2_^*a*^, *g*(*m*_*j*_ *a*) = *O*_2_^*a*^;
 then for ∀*m*_*i*_ ∈ *P*_1_^*a*^:
  (10) $$\begin{array}{c} g\left(m_{i}a\right)=O_{1}^{a}\neq O_{2}^{a}; \end{array}$$
 that is,
  (11) $$\begin{array}{c} m_{i}\notin P_{2}^{a}; \end{array}$$
 similarly, for ∀*m*_*j*_ ∈ *P*_2_^*a*^, *m*_*j*_ ∉ *P*_1_^*a*^;
 that is, *P*_*i*_^*a*^∩*P*_*j*_^*a*^ = Φ.

**Theorem 14 .**

For ∀*P*_*i*_^*a*^ ∈ *P* ^*a*^, satisfying *P*_*i*_^*a*^∩*F*(*a*) = Φ.

**Proof**

Suppose *P*_*i*_^*a*^∩*F*(*a*) ≠ Φ; that is, there exists element *m* satisfying *m* ∈ *P*_*i*_^*a*^ and*m* ∈ *F*(*a*):

 since *m* ∈ *F*(*a*),
 so
  (12) $$\begin{array}{c} g\left(am\right)=g\left(a\right); \end{array}$$
 according to Definition 11,
  (13) $$\begin{array}{c} g\left(am\right)=O_{i}^{a}\subset g\left(a\right); \end{array}$$
 obviously, Formulae (12) and (13) are mutually contradictory, so the assumption is not valid.
 So *P*_*i*_^*a*^∩*F*(*a*) = Φ.

In the formal context presented in Table 1, *F*(*d*) = {*g*}, *F*(*e*) = {*c*} are the father-attributes of child-attributes *d* and *e*, respectively.

As Definition 11 shows *P* ^*d*^ = Φ, while *e* is provided with *P* ^*e*^ ≠ Φ : *P*_1_^*e*^ = {*a*, *f*}, *O*_1_^*e*^ = {0,2}; *P*_2_^*e*^ = {*g*, *d*}, *O*_2_^*e*^ = {0,6}; and *P* ^*e*^ = {{*a*, *f*}, {*g*, *d*}}.

#### 3.2.2. Exploration of Vertex

(*1) Representation of the Path*. Suppose the set of all attributes in attribute topology is *X* = {*x*_1_, *x*_2_,…, *x*_*n*_}; that is, #{*X*} = *m*.

**Definition 15 .**

(Λ^*n*^ *X*, *∠*, *θ*) is triple which satisfies the following properties:

1. Λ^*n*^ *X* = *∠*(Λ^*n*^ *X*) · *θ* ^(Λ^*n*^*X*)^;
2. *∠*(Λ^*n*^ *X*) = *g*(*X*);
3. *θ* ^(Λ^*n*^*X*)^≜〈*x*_1_, *x*_2_,…, *x*_*n*_〉,

where Λ^*n*^ *X* satisfies the following:

1. Λ^*n*^ *X* = {*x*_1_Λ*x*_2_,…, Λ*x*_*n*_∣∀ *x*_*i*_ ∈ *X*, *i* ∈ [1, *n*]},
2. Λ^1^ *X* = {*x*_1_∣∀ *x*_1_ ∈ *X*},
3. *n* ≤ *m*,
4. {*x*_1_Λ*x*_2_}≠{*x*_2_Λ*x*_1_}, ∀*x*_1_, *x*_2_ ∈ *X*,
5. {{*x*_1_Λ*x*_2_}Λ*x*_3_} = {*x*_1_Λ{*x*_2_Λ*x*_3_}}, ∀*x*_1_, *x*_2_, *x*_3_ ∈ *X*,
6. {*x*_1_Λ*x*_2_Λ*x*_3_} = {*x*_1_Λ*x*_2_}Λ{*x*_2_Λ*x*_3_}, ∀*x*_1_, *x*_2_, *x*_3_ ∈ *X*.

As shown in Definition 15, *P* =  Λ^*n*^ *X* is uniquely determined by its magnitude and direction if #{*∠*(Λ^*n*^ *X*)} ≠ 0. Its magnitude is expressed by *∠P* = *∠*(Λ^*n*^ *X*) where *∠*(Λ^*n*^ *X*) = ⋂_*i*−1_^*n*^ *g*(*x*_*i*_) and its direction is expressed by *θ* ^*P*^ = *θ* ^Λ^*n*^*X*^.

According to the above analysis, the path can be represented by *P* = Λ^*n*^ *X*: the attributes successively passed by current path, and 〈*x*_1_, *x*_2_, *x*_3_,…, *x*_*n*_〉, are recorded by *θ* ^*P*^. There exists unidirectional edge between each two adjacent vertices; that is, edges passed by the current path are 〈*x*_1_, *x*_2_〉, 〈*x*_2_, *x*_3_〉,…, 〈*x*_*n*−1_, *x*_*n*_〉 successively. Let *∠P* be the weights on the edge 〈*x*_*n*−1_, *x*_*n*_〉.

Suppose adding a new vertex *x*_*n*+1_ to the existing path; then *P* = *P*Λ{*x*_*n*+1_}. Update on the path: a new vertex *x*_*n*+1_ and a new edge 〈*x*_*n*_, *x*_*n*+1_〉 are generated; that is, the attributes successively passed by the path are 〈*x*_1_, *x*_2_, *x*_3_,…, *x*_*n*_, *x*_*n*+1_〉 and the weights on the edge 〈*x*_*n*_, *x*_*n*+1_〉 are *∠P* = *∠*(Λ^*n*+1^ *X*).

**Theorem 16 .**

*X*_*n*_ = {*x*_1_, *x*_2_, *x*_3_,…, *x*_*n*_} must be a complete polygon in attribute topology if #{*∠*(Λ^*n*^ *X*)} ≠ 0.

**Proof**

Consider the following:

(14) $$\begin{array}{c} \#\left\{∠\left(\overset{n}{\Lambda }X\right)\right\}\neq 0. \end{array}$$

Then

(15) $$\begin{array}{c} ∠\left(\overset{n}{\Lambda }X\right)\neq \Phi ,\\ ∠\left(\overset{n}{\Lambda }X\right)=\overset{n}{\underset{i=1}{⋂}}g\left(x_{i}\right)\neq \Phi \end{array}$$

for ∀*x*_*k*_, *x*_*j*_ ∈ *X*

(16) $$\begin{array}{c} \overset{n}{\underset{i=1}{⋂}}g(x_{i})\subseteq g\left(x_{k}\right)\cap g\left(x_{j}\right) \end{array}$$

combined with Formulae (15) and (16), for ∀*x*_*k*_, *x*_*j*_ ∈ *X*

(17) $$\begin{array}{c} g\left(x_{k}\right)\cap g\left(x_{j}\right)\neq \Phi . \end{array}$$

So *X*_*n*_ = {*x*_1_, *x*_2_, *x*_3_,…, *x*_*n*_} must be a complete polygon.

**Lemma 17 .**

{*x*_1_, *x*_2_, *x*_3_,…, *x*_*n*_} not be the intension certainly if #{*∠*(Λ^*n*^ *X*)∣*n* < *m*} = 0.

**Proof**

Consider the following:

(18) $$\begin{array}{c} \#\left\{∠\left(\overset{n}{\Lambda }X\right)\;|\;n<m\right\}=0. \end{array}$$

Then

(19) $$\begin{array}{c} ∠\left(\overset{n}{\Lambda }X\right)=\Phi \end{array}$$

so

(20) $$\begin{array}{c} \overset{n}{\underset{i=1}{⋂}}g\left(x_{i}\right)=g\left(\overset{n}{\underset{i=1}{⋃}}x_{i}\right)=\Phi . \end{array}$$

To prove that {*x*_1_, *x*_2_, *x*_3_,…, *x*_*n*_} is the intension of a concept, satisfying Formulae (1) is needed; that is,

(21) $$\begin{array}{c} f\left(g\left(\overset{n}{\underset{i=1}{⋃}}x_{i}\right)\right)=\left\{\overset{n}{\underset{i=1}{⋃}}x_{i}\right\} \end{array}$$

since

(22) $$\begin{array}{c} f\left(g\left(\overset{n}{\underset{i=1}{⋃}}x_{i}\right)\right)=f\left(\Phi \right) \end{array}$$

in this formal context,

(23) $$\begin{array}{c} f\left(\Phi \right)=\left\{x_{1},x_{2},x_{3},\ldots ,x_{m}\right\} \end{array}$$

combined with Formulae (22) and (23):

(24) $$\begin{array}{c} f\left(g\left(\overset{n}{\underset{i=1}{⋃}}x_{i}\right)\right)=\left\{x_{1},x_{2},x_{3},\ldots ,x_{m}\right\}. \end{array}$$

Obviously, Formulae (21) and (24) are mutually contradictory; that is, {*x*_1_, *x*_2_, *x*_3_,…, *x*_*n*_} is not the intension.

**Lemma 18 .**

*X*′ = {*x*_1_, *x*_2_, *x*_3_,…, *x*_*n*−1_} must be the intension of a concept if #{*∠*(Λ^*n*−1^ *X*)} ≠ 0 and #_∀*x*_*s*_∈*X*−{*x*_1_,*x*_2_,…,*x*_*n*−1_}_{*∠*{(Λ^*n*−1^ *X*)Λ*x*_*s*_}} = 0.

**Proof**

Consider the following:

(25) $$\begin{array}{c} \#\left\{\overset{n-1}{\underset{i=1}{\Lambda }}x_{i}\;|\;x_{i}\in X\right\}\neq 0 \end{array}$$

 and then
  (26) $$\begin{array}{c} \overset{n}{\underset{i=1}{⋂}}g\left(x_{i}\right)=g\left(X^{\prime }\right)\neq \Phi \end{array}$$
 since #_∀*x*_*s*_∈*X*−{*x*_1_,*x*_2_,…,*x*_*n*−1_}_{*∠*{(Λ^*n*−1^ *X*)Λ*x*_*s*_}} = 0
 so for ∀*x*_*s*_ ∈ *X* − *X*′,
  (27) $$\begin{array}{c} g\left(X^{\prime }\right)\cap g\left(x_{s}\right)=\Phi . \end{array}$$

According to Formulae (1), to prove the intension of a formal concept, it is only needed to satisfy *f*(*g*(*X*′)) = *X*′. From the relationship of formal context,

(28) $$\begin{array}{c} X^{\prime }\subseteq f\left(g\left(X^{\prime }\right)\right) \end{array}$$

if *X*′ ⊂ *f*(*g*(*X*′)); then there must be

(29) $$\begin{array}{c} f\left(g\left(X^{\prime }\right)\right)=X^{\prime }\cup N, \end{array}$$

where *N*⊆*X* − *X*′ and *N* ≠ Φ, and then

(30) $$\begin{array}{c} g\left(f\left(g\left(X^{\prime }\right)\right)\right)=g\left(X^{\prime }\cup N\right)=g\left(X^{\prime }\right)\cap g\left(N\right). \end{array}$$

Also

(31) $$\begin{array}{c} g\left(f\left(g\left(X^{\prime }\right)\right)\right)=g\left(X^{\prime }\right). \end{array}$$

Simultaneous Formulae (30) and (31) will get

(32) $$\begin{array}{c} g\left(X^{\prime }\right)=g\left(X^{\prime }\right)\cap g\left(N\right). \end{array}$$

Combined with Formulae (27) and (32):

(33) $$\begin{array}{c} g\left(X^{\prime }\right)=\Phi \end{array}$$

apparently the result is wrong.

So

(34) $$\begin{array}{c} X^{\prime }⊄f\left(g\left(X^{\prime }\right)\right),\\ f\left(g\left(X^{\prime }\right)\right)=X^{\prime }. \end{array}$$

So *X*′ is the intension of a formal concept.

(*2) Process of Depth Exploration of Vertex*. Depth exploration of vertices in topology is essentially the process of exploring the adjacent points through the arcs between vertices. Suppose the set of all attributes of formal context is *X*  (#*X* = *n* − 2), and the sets *A* and *B* are the sets of top-attributes and child-attributes, respectively. According to Section 3.2.1(1), the set of vertices sorted is *X*_(*A*,*B*)_^*T*^ = {*x*_1_, *x*_2_,…, *x*_*n*_}, based on which to explore vertices.

This algorithm begins with the first element, that is, start point Ψ, and then explores the subsequent elements successively. For the sorted set *X*_(*A*,*B*)_^*T*^, *x*_2_ = Next(Ψ), *x*_3_ = Next(*x*_2_).

**Figure 3:**
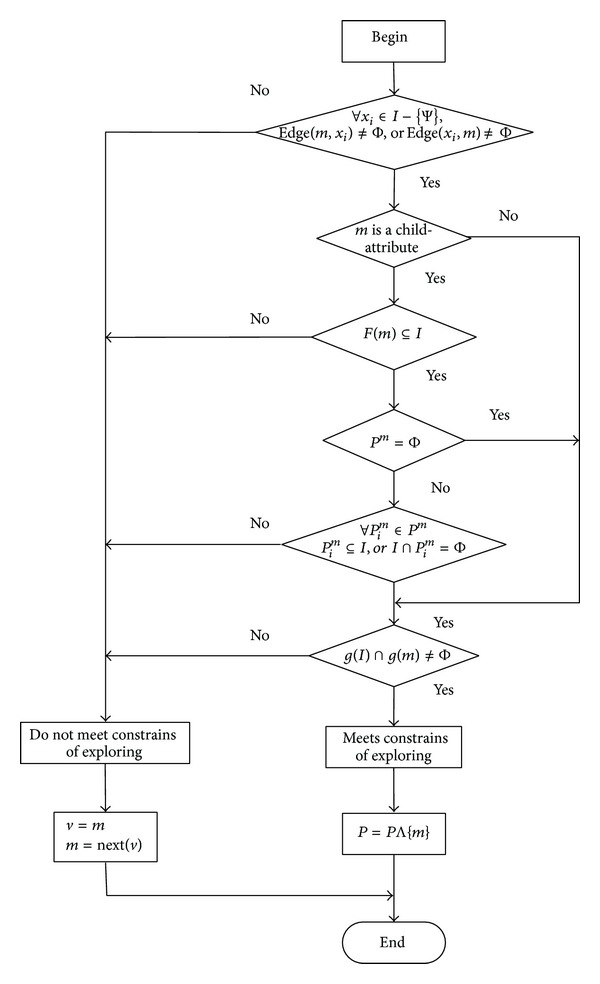
Flowchart of the process of exploring attribute *m* and the constrains related.

Suppose *P* = Λ^*k*−1^ *X*_(*A*,*B*)_^*T*^; *k* ≤ *n* is the current path and ordered set *I* = {*x*_1_, *x*_2_,…, *x*_*k*−1_} is the set of attributes which passed by the path successively. Suppose that attribute *m* is the current attribute explored, and the process of exploring *m* and the constrains of exploring are plotted in Figure 3.

As seen in Figure 3, when exploring the attribute *m*, constraints are shown as follows:

1. ∀*x*_*i*_ ∈ *I* − {Ψ}, Edge(*m*, *x*_*i*_) ≠ Φ, or Edge(*x*_*i*_, *m*) ≠ Φ;
2. *m* is a child-attribute;
3. *F*(*m*)⊆*I*;
4. *P* ^*m*^ = Φ;
5. for ∀*P*_*i*_^*m*^ ∈ *P* ^*m*^, satisfying *P*_*i*_^*m*^⊆*I*, or *I*∩*P*_*i*_^*m*^ = Φ;
6. *g*(*I*)∩*g*(*m*) ≠ Φ.

Figure 3 displays that the current path is updated, that is, *P* = *P*Λ{*m*} and *I* = *I* ∪ {*m*}, when it meets any one of the following conditions, that is, the constrains of exploring:

- $(1)\cap \bar{\left(2\right)}\cap (6)=\text{true}$;
- (1)∩(2)∩(3)∩(4)∩(6) = true;
- $(1)\cap (2)\cap (3)\cap \bar{\left(4\right)}\cap (5)\cap (6)=\text{true}$.

It is needed to traverse the attribute next to attribute (*v* = *m*, *m* = Next(*v*)), when it meets the condition $\bar{a}\;\cap \;\bar{b}\;\cap \;\bar{c}=\text{true}$, that is, not satisfying the constrains of exploring.

Then we analyze the exploring process through an example. For the formal context shown in Table 1, *X*_(*A*,*B*)_^*T*^ = {Ψ, *a*, *b*, *c*, *g*, *f*, *d*, *e*, *E*}.

This algorithm begins with the start point, that is, *P* = {Ψ}, *I* = {Ψ}; current attribute explored *m* = *a*. Attribute *a* satisfies the following conditions: *a* is not the child-attribute; *g*(*I*)∩*g*(*a*) ≠ Φ; that is, attribute *a* meets the above condition (a). The current path is updated:*P* = {ΨΛ*a*}, *I* = {Ψ, *a*}; the weights on the newly added edge 〈Ψ, *a*〉 are *w* 〈Ψ, *a*〉 = *∠P* = *g*(Ψ)∩*g*(*a*) = *g*(*a*) = {0,2, 3,5, 7,8}; and the direction of the path updated is *θ* ^*P*^ = 〈Ψ, *a*〉, that is, from Ψ to *a*.

**Figure 4:**
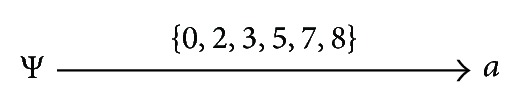
The updated path from the path *P* = {Ψ}.

The process above can be demonstrated by Figure 4.

Figure 4 displays that the path = {ΨΛ*a*}: *θ* ^*P*^ is presented as the arrow shown; *∠P*, as the weights, is marked on the newly generated edge.

Additionally, suppose *P* = {ΨΛ*a*Λ*b*Λ*c*}; *I* = {Ψ, *a*, *b*, *c*} is the current path, and current attribute explored *m* = *e*. Attribute *e* satisfies the following conditions:

1. ∀*x*_*i*_ ∈ *I* − {Ψ}, Edge(*x*_*i*_, *e*) ≠ Φ;
2. *e* is a child-attribute;
3. *F*(*e*)⊆*I*(*F*(*e*) = {*c*});
4. *P* ^*e*^ = Φ(*P*_1_^*e*^ = {*a*, *f*}, *P*_2_^*e*^ = {*d*, *g*});
5. *P*_1_^*e*^⊄*I*, *I*∩*P*_2_^*e*^ = Φ.

That is, *e* satisfies the above condition $\bar{a}\;\cap \;\bar{b}\;\cap \;\bar{c}=\text{true}$; then it explores the attribute *E* next to attribute *e*(Next(*e*) = *E*): when the current attribute explored *m* is updated from *e* to *E*, the current path does not update.

(*3) Updated Data in Process of Depth Exploration of Vertex*. From the previous section analysis, the path is updated when the explored attribute *m* meets the constrains of exploring and adds to the current path. There are a series of data to be updated, mainly in the following two aspects.

(*1) Updated Set of Concepts C*. Suppose the set of all attributes of formal context is *X*  (#*X* = *n* − 2), *X*_(*A*,*B*)_^*T*^ = {*x*_1_, *x*_2_,…, *x*_*n*_}, #*X*_(*A*,*B*)_^*T*^ = *n*. Let the set of attribute category be {0,1} and the category of attribute be Mark(*x*_*i*_), that is, Mark(*x*_*i*_)∈{0,1}. For ∀*x*_*i*_ ∈ *X*_(*A*,*B*)_^*T*^, Mark(*x*_*i*_) is initialized to 0. The set of concepts *C* is initialized to Φ.

Suppose the current path is *P* = Λ^*k*^ *X*_(*A*,*B*)_^*T*^, *k* ≤ *n*, *I* = {*x*_1_, *x*_2_,…, *x*_*k*_}, and the current set of concepts is *C* = {*c*_1_, *c*_2_,…, *c*_*l*_}, and for ∀*c*_*j*_ ∈ *C*, *c*_*j*_ = (*A*_*j*_, *B*_*j*_), that is, *C* = {(*A*_1_, *B*_1_), (*A*_2_, *B*_2_),…, (*A*_*l*_, *B*_*l*_)}.

Suppose the current attribute explored *m* satisfies the constrains of exploring shown in previous section and the path is updated, that is, *P*′ = *P*Λ{*m*}, *∠P*′ = *∠P*∩*g*(*m*), *I*′ = *I* ∪ {*m*}.

Simultaneously, the set *C* is updated.

If *∠P*′ = *∠P*, let the newly generated two-tuples (*∠P*′, *I*′)→(*A*_*s*_, *B*_*s*_) : *A*_*s*_ = *∠P*, *s* ≤ *l*. That is, the set *C* is updated to *C* = {(*A*_1_, *B*_1_), (*A*_2_, *B*_2_),…, (*A*_*l*−1_, *B*_*l*−1_)(*∠P*′, *I*′)}, *s* = *l* or *C* = {(*A*_1_, *B*_1_), (*A*_2_, *B*_2_),…, (*A*_*s*−1_, *B*_*s*−1_)(*∠P*′, *I*′),…, (*A*_*s*_, *B*_*s*_)}, *s* < *l*; Mark(*x*_*k*_) = 1.

If *∠P*′ ≠ *∠P*, *C* = *C* ∪ {(*∠P*′, *I*′)}, that is, *C* = {(*A*_1_, *B*_1_), (*A*_2_, *B*_2_),…, (*A*_*l*_, *B*_*l*_)(*∠P*′, *I*′)}.

(*2) Updated Attribute Topology.* If *∠P*′ = Edge(*x*_*k*_, *m*), then let Edge(*x*_*k*_, *m*) = Edge(*m*, *x*_*k*_) = Φ; that is, the edge (unidirectional or bidirectional), between *x*_*k*_ and *m*, is removed from the attribute topology.

If *∠P*′ ≠ Edge(*x*_*k*_, *m*), the attribute remains unchanged.

On the contrary, updates on the two aspects described above will not be induced if the current attribute explored *m* does not meet the constrains of exploring shown in previous section.

Then we analyze the date updated through an example.

**Figure 5:**
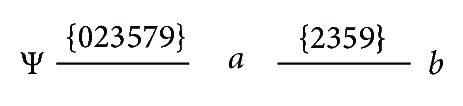
The path updated from the path listed in Figure 4.

For the formal context shown in Table 1, suppose the current path is *P* = {ΨΛ*a*} and *I* = {Ψ, *a*}, *C* = {({0,1, 2,3, 4,5, 6,7, 8}, {Ψ})({0,2, 3,5, 7,8}, {Ψ, *a*})}, which is presented in Figure 4. Suppose the current attribute explores *m* = *b*. Attribute *b* meets the constrains of exploring shown in previous section and path is updated: *P*′ = {ΨΛ*a*Λ*b*}, *∠P*′ = *g*(Ψ*a*)∩*g*(*b*) = {0,2, 3,5, 7,8}∩{2,3, 4,5, 6,8} = {2,3, 5,8}, and *I*′ = {Ψ, *a*, *b*}. The updated path is demonstrated by Figure 5, and for convenience of description, the newly generated edge is presented by the route without an arrow.

The updated data is shown as follows. 

*Updated Set C*. *∠P*′ = {2,3, 5,8} ≠ *∠P* = {0,2, 3,5, 7,8} and then adds the newly generated two-tuples ({2,3, 5,8}, {Ψ, *a*, *b*}) to the set *C*, that is, *C* = {({0,1, 2,3, 4,5, 6,7, 8}, {Ψ}), ({0,2, 3,5, 7,8}, {Ψ, *a*}),   ({2,3, 5,8}, {Ψ, *a*, *b*})}. The update on the set of concepts *C*, along with the update on path, is shown in Table 2.

*Updated Attribute Topology*. Suppose *∠P*′ = {2,3, 5,8}. Figure 2 presents that Edge(*a*, *b*) = {2,3, 5,8} which satisfies the condition *∠P*′ = Edge(*x*_*k*_, *m*), so let Edge(*a*, *b*) = Edge(*b*, *a*) = Φ; that is, the bidirectional edge, between *a* and *b*, is removed from the attribute topology.

**Figure 6:**
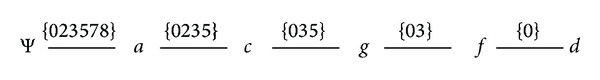
The current path *P* = {ΨΛ*a*Λ*c*Λ*g*Λ*f*Λ*d*}.

Additionally, Suppose *P* = {ΨΛ*a*Λ*c*Λ*g*Λ*f*Λ*d*}, listed in Figure 6, is the current path and *C* = {({0,1, 2,3, 4,5, 6,7, 8}, {Ψ}),…, ({0}, {Ψ, *a*, *c*, *g*, *f*, *d*})}, *I* = {Ψ, *a*, *c*, *g*, *f*, *d*}.

**Figure 7:**
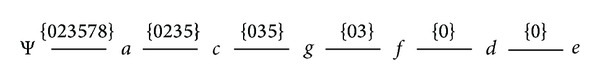
The path updated from the path listed in Figure 6.

Suppose the current attribute explored *m* = *e*. Attribute *e* satisfies the constrains of exploring and then the path is updated: *P* = {ΨΛ*a*Λ*c*Λ*g*Λ*f*Λ*d*Λ*e*}, *∠P*′ = *∠P*∩*g*(*b*) = {0,3}∩{0,4, 5,6, 8} = {0}. The updated path is presented in Figure 7.

The updated data is shown as the following.

*Updated Set C*. Suppose *∠P*′ = {0} = *∠P* = {0}. Then the element ({0}, {Ψ, *a*, *c*, *g*, *f*, *d*, *e*}) in set *C* is replaced with the newly generated two-tuples ({0}, {Ψ, *a*, *c*, *g*, *f*, *d*, *e*}); that is, the number of elements in set *C* remains unchanged: Mark(*d*) = 1.

Table 3 lists each update on set *C*, along with each update on path, from path *P* = {Ψ} to the current path *P* = {ΨΛ*a*Λ*c*Λ*g*Λ*f*Λ*d*Λ*e*}.

*Updated Attribute Topology*. Suppose *∠P*′ = {0}. Figure 2 presents that Edge(*d*, *e*) = {0,6} which satisfies the condition *∠P*′ ≠ Edge(*x*_*k*_, *m*), so the attribute topology remains unchanged.

#### 3.2.3. Backtracking of Vertex

Suppose the set of all attributes of formal context is *X*  (#*X* = *n* − 2), *X*_(*A*,*B*)_^*T*^ = {*x*_1_, *x*_2_,…, *x*_*n*_}, #*X*_(*A*,*B*)_^*T*^ = *n* and the current path is *P* = Λ^*k*^ *X*_(*A*,*B*)_^*T*^, *k* ≤ *n*, *I* = {*x*_1_, *x*_2_,…, *x*_*k*_}.

Backtracking the vertex occurs if the current attribute explored *m* satisfies any of the following two conditions, that is, constrains of backtracking:

1. *m* = *E*  &  *P* ≠ {Ψ};
2. Mark(*m*) = 1.

Suppose the current path is *P* =  Λ^*k*^ *X*_(*A*,*B*)_^*T*^, *k* ≤ *n*, and *I* = {*x*_1_, *x*_2_,…, *x*_*k*_}. When the current attribute explored *m* satisfies the constrains of backtracking, backtracking the vertex: *P*′ = Λ^*k*−1^ *X*_(*A*,*B*)_^*T*^, that is, *P*′ = {*x*_1_Λ*x*_2_Λ,…, *x*_*k*−1_}, *∠P*′ = ⋂_*i*=1_^*k*−1^ *g*(*x*_*i*_), *I*′ = {*x*_1_, *x*_2_,…, *x*_*k*−1_}, Mark(*x*_*k*_) = 0, and set *C* remains unchanged.

**Figure 8:**
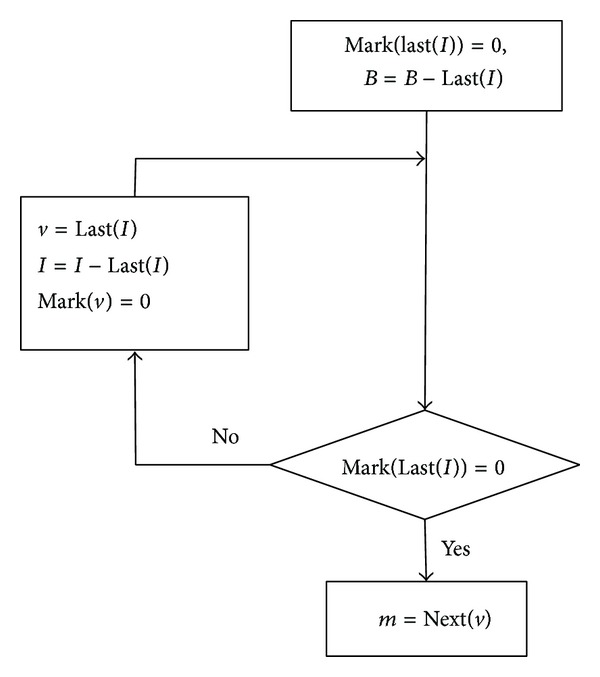
Flowchart of the process of backtracking vertices.

Combined with the description above, the process of backtracking vertices is listed in Figure 8. Last(·) represents the last element of the order set.

The process of backtracking vertices is finished when it meets the condition $\bar{\left(1\right)}\cap \bar{\left(2\right)}=\text{true}$, according to Figure 8.

Then, the process of backtracking vertices is illustrated by the example.

Suppose the current path is shown in Figure 7. According to Section 3.2.2, Mark(⋃_*i*=1_^7^ *x*_*i*_) = {0,0, 0,0, 0,1, 0}, Mark(*d*) = 1.

Suppose the current attribute explored *m* = *E*, which satisfies the constrains of backtracking, then it backtracks the vertex, that is, *P* = {ΨΛ*a*Λ*c*Λ*g*Λ*f*Λ*d*}, *I* = {Ψ, *a*, *c*, *g*, *f*, *d*}, Mark(*e*) = 0.

**Figure 9:**
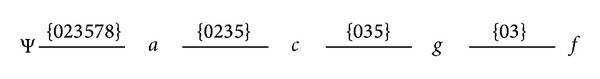
The path updated from the path listed in Figure 7.

Then a judge on Mark(Last(*I*)) is achieved, presented in Figure 9. Due to the fact that Mark(Last(*I*)) = Mark(*d*) = 1 satisfies the constrains of backtracking, backtracking of vertex is achieved again, that is, *P* = {ΨΛ*a*Λ*c*Λ*g*Λ*f*}, *I* = {Ψ, *a*, *c*, *g*, *f*}, Mark(*d*) = 0 and *v* = *d*.

Repeating the judge on Mark(Last(*I*)), and Mark(Last(*I*)) = Mark(*f*) = 0, which leads to the completion of the backtracking vertices. Then the current attribute explored *m* is updated to Next(*v*) = Next(*d*) = *e* and the set *C* remains unchanged. The updated path is shown in Figure 9.

#### 3.2.4. Discussion

**Figure 10:**
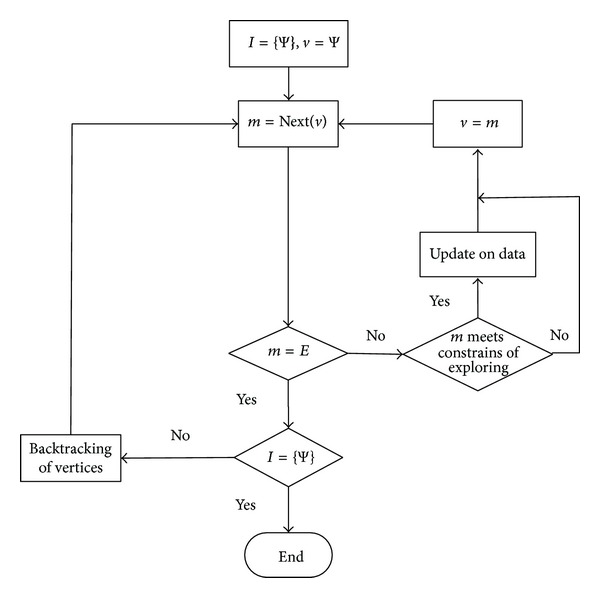
Flowchart of the complete algorithm.

Flowchart of the complete algorithm is listed in Figure 10.

As seen in Figure 10, the process of exploring *m* is achieved when the backtracking process is finished. In the process of depth exploration, the process of backtracking *m* is achieved if it satisfies the constrains of backtracking. That is, all paths between the two fixed points which meet related constrains are achieved in the process of repeating, exploring, and backtracking vertices. The update on the set of concepts *C* and attribute topology are induced in the process of traversing the path.

Figure 10 presents that the algorithm ends when it meets the condition (*m* = *E*)∩(*P* = {Ψ}) = true.

**Figure 11:**
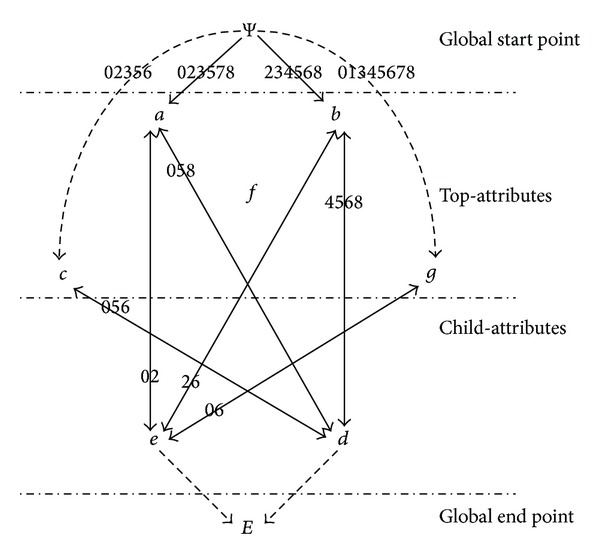
The updated attribute topology after the end of the algorithm.

The update on set *C* and attribute topology, after the end of the algorithm, are demonstrated, respectively, by the example presented above:

1. updated topology is shown in Figure 11;
2. updated set *C*.

Each two-tuples ({Ψ} ∪ *A*, *B*) is updated to (*A*, *B*) due to the fact that the start point Ψ is not of practical significance. Then every element in set *C* is a concept; that is, *β*(*G*, *M*, *I*) = *C* ∪ {(Φ, *abcdefg*)} in formal context *K*≔(*G*, *M*, *I*).

**Figure 12:**
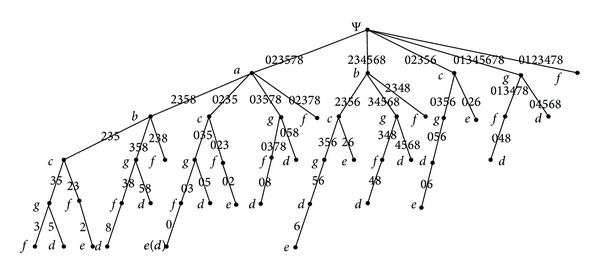
The path traversal tree of the attribute topology presented in Figure 2.

The traversal process of all paths between the start and end points is illustrated by the tree structure shown in Figure 12.

Figure 12 displays that each path begins with Ψ and ends with a leaf node. Each path is generated from top to bottom and all paths are formed from left to right.

Any node *n* in Figure 12 can be seen as a concept, with the set of all attributes passed by the current path in which current node *n* located, from Ψ to node *n*, as the intension and the weights on the edge, whose lower end is connected to the current node *n*, as the extension. The node *e*(*d*) presents that attribute *d* is also passed by the current path where node *n* located.

**Figure 13:**
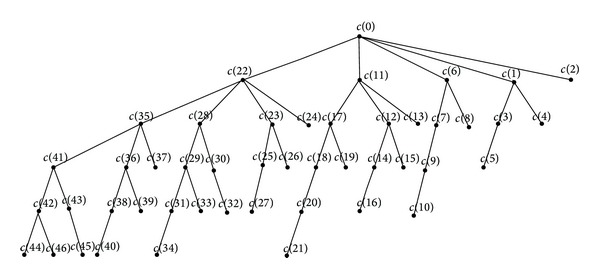
The concept tree of the attribute topology presented in Figure 2.

As seen in Figure 13, the concepts represented by nodes in Figure 12 are indicated on the corresponding nodes and generated a concept tree. Figure 13 displays all concepts except the global concept (Φ, *abcdefg*).

**Figure 14:**
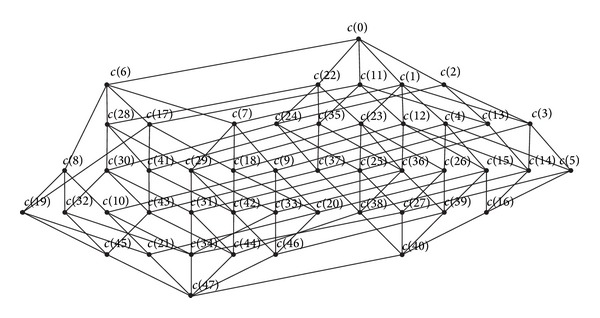
Hasse diagram of the formal context presented in Table 1.

Figure 14 shows the Hasse diagram of the formal context plotted in Table 1 and each node represents a formal concept.

The concepts successively represented by c(0)–c(47) are (012345678,ø), (01345678,*g*), (0123478,*f*), (013478,*gf*), (04568,*gd*), (048,*gfd*), (02356,*c*), (0356,*cg*), (026,*ce*), (056,*cgd*), (06,*cgde*), (234568,*b*), (34568,*bg*), (2348,*bf*), (348,*bgf*), (4568,*bgd*), (48,*bdfg*), (2356,*bc*), (356,*bcg*), (6,*bce*), (56,*bcgd*), (6,*bcgde*), (023578,*a*), (03578,*ag*), (02378,*af*), (0378,*agf*), (058,*agd*), (08,*agfd*), (0235,*ac*), (035,*acg*), (023,*acf*), (03,*acgf*), (02,*acfe*), (05,*acgd*), (0,*acgfde*), (2358,*ab*), (258,*abg*), (238,*abf*), (38,*abgf*), (58,*abgd*), (8,*abgfd*), (235,*abc*), (35,*abcg*), (23,*abcf*), (3,*abcgf*), (2,*abcgfe*), (5,*abcgd*), and (ø,*abcdefg*), that is, all concepts in the formal context.

Under the constrains and calculation rules, the process of computing all formal concepts is achieved by traversing vertices successively. The path is formed by the traversed vertices and all concepts are induced in the process of traversing all paths. As shown in Figures 12 and 13, the process of traversing all paths is represented by the concept tree, demonstrating the calculation of all the concepts intuitively and vividly.

As seen in Figure 13, all concepts are achieved completely and the calculation process is presented intuitively, according to the global formal concepts searching of attribute topology. Comparing Figures 13 and 14, as the part of Hasse diagram, the concept tree not only demonstrates the hierarchical structures among concepts clearly but is also much simpler than the Hasse diagram.

## 4. Conclusion

Global formal concepts searching of attribute topology is proposed in this paper based on the concepts of calculating with subtopologies. With the basic idea of Depth First Search, this algorithm, beginning with the global start point, employs the constraints and calculation rules to explore and backtrack the attributes of the degenerated topology repeatedly until traversal of all paths is achieved. The set of concepts is updated throughout the process and all concepts are obtained ultimately. This method avoids the decomposition process of the whole topology, which reflects the integrity of the algorithm. Visualization features of the calculation process are enhanced by the concept tree. This method makes the whole process more logical and feasible, easy to implement, and suitable for large-scale data sets. Attribute topology provides a new approach of representation of the formal context. The further step is to refine and optimize the method and put it into application.

## Figures and Tables

**Table 1:** A formal context that contains child-attributes.

	*a*	*b*	*c*	*d*	*e*	*f*	*g*
0	1	0	1	1	1	1	1
1	0	0	0	0	0	1	1
2	1	1	1	0	1	1	0
3	1	1	1	0	0	1	1
4	0	1	0	1	0	1	1
5	1	1	1	1	0	0	1
6	0	1	1	1	1	0	1
7	1	0	0	0	0	1	1
8	1	1	0	1	0	1	1

**Table 2:** The update on set *C*, along with the update on the path shown in Figure 5.

*P*	*C*
{ΨΛ*a*}	({0, 1, 2, 3, 4, 5, 6, 7, 8}, {Ψ})
	({0, 2, 3, 5, 7, 8}, {Ψ, *a*})

{ΨΛ*a*Λ*b*}	({0, 1, 2, 3, 4, 5, 6, 7, 8}, {Ψ})
	({0, 2, 3, 5, 7, 8}, {Ψ, *a*})
	({2, 3, 5, 8}, {Ψ, *a*, *b*})

**Table 3:** The update on set *C*, along with the update on the path shown in Figure 7.

*P*	*C*
{Ψ}	({0, 1, 2, 3, 4, 5, 6, 7, 8}, {Ψ})

{ΨΛ*a*}	({0, 1, 2, 3, 4, 5, 6, 7, 8}, {Ψ})
	({0, 2, 3, 5, 7, 8}, {Ψ, *a*})

⋮	⋮

{ΨΛ*a*Λ*c*Λ*g*Λ*f*Λ*d*}	({0, 1, 2, 3, 4, 5, 6, 7, 8}, {Ψ})
	⋮
	({0, 3}, {Ψ, *a*, *c*, *g*, *f*})
	({0}, {Ψ, *a*, *c*, *g*, *f*, *d*})

{ΨΛ*a*Λ*c*Λ*g*Λ*f*Λ*d*Λ*e*}	({0, 1, 2, 3, 4, 5, 6, 7, 8}, {Ψ})
	⋮
	({0, 3}, {Ψ, *a*, *c*, *g*, *f*})
	({0}, {Ψ, *a*, *c*, *g*, *f*, *d*, *e*})
